# Supplementary material for: Autophagy-regulated mitochondrial inheritance controls early CD8+ T cell fate commitment
Source: Nat Cell Biol. 2025 Dec 19;28(1):66–81. doi: 10.1038/s41556-025-01835-2 (PMC12807857; doi:10.1038/s41556-025-01835-2)
Supplement: Supplementary file 1 — Reporting Summary [file 41556_2025_1835_MOESM1_ESM.pdf]

nature portfolio

Corresponding author(s): Mariana Borsa
Anna Katharina Simon

Last updated by author(s): 23/10/25

Reporting Summary

Nature Portfolio wishes to improve the reproducibility of the work that we publish. This form provides structure for consistency and transparency in reporting. For further information on Nature Portfolio policies, see our Editorial Policies and the Editorial Policy Checklist.

Statistics

For all statistical analyses, confirm that the following items are present in the figure legend, table legend, main text, or Methods section.

n/a	Confirmed	
☐	☒	The exact sample size (*n*) for each experimental group/condition, given as a discrete number and unit of measurement
☐	☒	A statement on whether measurements were taken from distinct samples or whether the same sample was measured repeatedly
☐	☒	The statistical test(s) used AND whether they are one- or two-sided<br>*Only common tests should be described solely by name; describe more complex techniques in the Methods section.*
☒	☐	A description of all covariates tested
☐	☒	A description of any assumptions or corrections, such as tests of normality and adjustment for multiple comparisons
☐	☒	A full description of the statistical parameters including central tendency (e.g. means) or other basic estimates (e.g. regression coefficient) AND variation (e.g. standard deviation) or associated estimates of uncertainty (e.g. confidence intervals)
☒	☐	For null hypothesis testing, the test statistic (e.g. *F*, *t*, *r*) with confidence intervals, effect sizes, degrees of freedom and *P* value noted<br>*Give P values as exact values whenever suitable.*
☒	☐	For Bayesian analysis, information on the choice of priors and Markov chain Monte Carlo settings
☒	☐	For hierarchical and complex designs, identification of the appropriate level for tests and full reporting of outcomes
☒	☐	Estimates of effect sizes (e.g. Cohen's *d*, Pearson's *r*), indicating how they were calculated

Our web collection on statistics for biologists contains articles on many of the points above.

Software and code

Policy information about availability of computer code

Data collection	Data were collected using: BD FACSDiva (Fortessa, LSR or ARIA III hardware), ZenBlue or Olympus Software (ZEISS 980 Airyscan 2, Olympus IX83 inverted microscope), Seahorse Software.
Data analysis	Flow cytometry data were analyzed by Flowjo 10.10.0. Microscopy analysis was performed using ImageJ software. Proteomics data was analyzed using Excel datasheets and visualized in Tableau. scRNAseq was analyzed using R software and visualized in Loupe Software. Mitochondria segmentation imaging analysis was done using scikit-image (Python). Data was exported into Graphpad prism (version 10.4.2) for statistical analysis.

For manuscripts utilizing custom algorithms or software that are central to the research but not yet described in published literature, software must be made available to editors and reviewers. We strongly encourage code deposition in a community repository (e.g. GitHub). See the Nature Portfolio guidelines for submitting code & software for further information.

Data

Policy information about availability of data

All manuscripts must include a data availability statement. This statement should provide the following information, where applicable:

- Accession codes, unique identifiers, or web links for publicly available datasets
- A description of any restrictions on data availability
- For clinical datasets or third party data, please ensure that the statement adheres to our policy

The mass spectrometry proteomics data have been deposited to the ProteomeXchage Consortium via the PRIDE partner repository with the dataset identifier PXD053316. Single cell transcriptomics data have been deposited at GEO (GSE270704). Metabolomics data are found in the Source Data File. Additional flow cytometry and imaging data supporting the findings are available from the first/corresponding author upon request.

Research involving human participants, their data, or biological material

Policy information about studies with human participants or human data. See also policy information about sex, gender (identity/presentation), and sexual orientation and race, ethnicity and racism.

Reporting on sex and gender	*Use the terms sex (biological attribute) and gender (shaped by social and cultural circumstances) carefully in order to avoid confusing both terms. Indicate if findings apply to only one sex or gender; describe whether sex and gender were considered in study design; whether sex and/or gender was determined based on self-reporting or assigned and methods used.<br>Provide in the source data disaggregated sex and gender data, where this information has been collected, and if consent has been obtained for sharing of individual-level data; provide overall numbers in this Reporting Summary. Please state if this information has not been collected.<br>Report sex- and gender-based analyses where performed, justify reasons for lack of sex- and gender-based analysis.*
Reporting on race, ethnicity, or other socially relevant groupings	*Please specify the socially constructed or socially relevant categorization variable(s) used in your manuscript and explain why they were used. Please note that such variables should not be used as proxies for other socially constructed/relevant variables (for example, race or ethnicity should not be used as a proxy for socioeconomic status).<br>Provide clear definitions of the relevant terms used, how they were provided (by the participants/respondents, the researchers, or third parties), and the method(s) used to classify people into the different categories (e.g. self-report, census or administrative data, social media data, etc.)<br>Please provide details about how you controlled for confounding variables in your analyses.*
Population characteristics	*Describe the covariate-relevant population characteristics of the human research participants (e.g. age, genotypic information, past and current diagnosis and treatment categories). If you filled out the behavioural & social sciences study design questions and have nothing to add here, write "See above."*
Recruitment	*Describe how participants were recruited. Outline any potential self-selection bias or other biases that may be present and how these are likely to impact results.*
Ethics oversight	*Identify the organization(s) that approved the study protocol.*

Note that full information on the approval of the study protocol must also be provided in the manuscript.

Field-specific reporting

Please select the one below that is the best fit for your research. If you are not sure, read the appropriate sections before making your selection.

☒ Life sciences ☐ Behavioural & social sciences ☐ Ecological, evolutionary & environmental sciences

For a reference copy of the document with all sections, see nature.com/documents/nr-reporting-summary-flat.pdf

Life sciences study design

All studies must disclose on these points even when the disclosure is negative.

Sample size	For microscopy analysis of dividing cells, the totality of mitotic cells found in each sample were imaged/acquired. The numbers varied depending on the abundance of the subsets analyzed, their proliferation potential, and the quality of the preparation prior to imaging. For analysis of mitochondrial geometric parameters, high resolution Z-stack images of >30 cells were analyzed. For functional readouts, 3-5 recipient mice were used per group within each experiment. This number was based on previous experience with adoptive transfers of T cells followed by acute infections (Borsa et al., Science Immunology 2019), the availability of the mice, and feasibility of the experiment. For in vitro assays (e.g. metabolic reliance, survival, proliferation, phenotyping), cells from at least 3 mice were used in independent experiments with technical replicates. Proteomics experiments had cells from at least 6 different mice contributing to the results (minimum of 2 samples, each representing a pool of 2-3 mice). For scRNAseq experiments, cells from 5 mice were used and pooling was done just prior to encapsulation for library preparation. For metabolomics experiments, cells from 3-5 mice were used as independent biological replicates. No statistical test was used to predetermine sample size.
Data exclusions	Proteomics data from CD8hi and CD8lo cells had samples excluded following analysis of histone counts.

Replication	In vitro experiments and in vivo readouts represented in main figures were repeated at least 2x. Rare exceptions include screening imaging results or those where alternative methods were used to address the same question. When applicable, this is made clear in figure legends. All replication attempts were successful, but pooling of the data was not always done in the graphs because of differences in cell survival and proliferation potential across experiments or due to variation of fluorescence intensity when Mean Fluorescent Intensity (MFI) was the main readout.
Randomization	Recipient mice were randomly distributed amongst groups at the start of each experiment to avoid age and sex bias.
Blinding	For in vivo experiments it is required by local authorities to state on the cage cards all handling that is done to the mice. Investigators were not blinded during data collection and analysis of freshly isolated tissues or cultured cells. When possible, unbiased sample processing and analysis were performed (proteomics, transcriptomics, metabolomics and part of imaging analysis).

Reporting for specific materials, systems and methods

We require information from authors about some types of materials, experimental systems and methods used in many studies. Here, indicate whether each material, system or method listed is relevant to your study. If you are not sure if a list item applies to your research, read the appropriate section before selecting a response.

Materials & experimental systems

n/a	Involved in the study	
☐	☒	Antibodies
☒	☐	Eukaryotic cell lines
☒	☐	Palaeontology and archaeology
☐	☒	Animals and other organisms
☒	☐	Clinical data
☒	☐	Dual use research of concern
☒	☐	Plants

Methods

n/a	Involved in the study	
☒	☐	ChIP-seq
☐	☒	Flow cytometry
☒	☐	MRI-based neuroimaging

Antibodies

Antibodies used

T cell activation:
anti-CD3 145-2C11 (BioLegend, 100340)
anti-CD28 37.51 (BioLegend, 102116)

Microscopy:
mouse anti-β-tubulin (Sigma-Aldrich, T8328)
mouse anti-Tom20 (F-10) (SantaCruz, sc-17764)
rabbit anti-MTHFD2 (EPR26938-20) (Abcam, ab307428)
rabbit anti-SHMT2 (E7X5B) (Cell Signaling, #93566)anti-CD8 APC (53-6.7) (BioLegend, 100712)
anti-mouse IgG AF488 (Abcam, ab150113)
anti-rabbit IgG AF594 (Invitrogen, A-11011)
anti-LC3B (D11) XP® Rabbit mAb PE (Cell Signaling, #3868)
anti-Tomm20 AF405 (EPR15581-54) (Abcam, ab210047)

Flow Cytometry and FACS:
anti-CD11b N418 PE-Cy7 (Biolegend, 117318)
anti-CD127 A7R34 BV711 (Biolegend, 135035)
anti-CD127 A7R34 BV785 (Biolegend, 135037)
anti-CD127 A7R34 PerCPCy5.5 (eBioscience, 45-1271-82)
anti-CD25 PC61 AF700 (Biolegend, 102024)
anti-CD25 PC61 PECy7 (Biolegend, 102015)
anti-CD25 PC61 APC (Biolegend, 102012)
anti-CD44 IM7 AF700 (Biolegend, 103026)
anti-CD44 IM7 BV785 (Biolegend, 103041)
anti-CD44 IM7 PE (Biolegend, 103008)
anti-CD44 IM7 PerCPCy5.5 (Biolegend, 103032)
anti-CD45.1 A20 BV785 (Biolegend, 110743)
anti-CD45.1 A20 FITC (Biolegend, 110706)
anti-CD45.1 A20 PB (Biolegend, 110722)
anti-CD45.2 104 AF700 (Biolegend, 109822)
anti-CD45.2 104 BV711 (Biolegend, 109847)
anti-CD45.2 104 FITC (Biolegend, 109806)
anti-CD62L MEL-14 FITC (Biolegend, 104406)
anti-CD62L MEL-14 eF450 (eBioscience, 48-0621-82)
anti-KLRG1 2F1 BV711 (Biolegend, 138427)
anti-KLRG1 BV785 (Biolegend, 138429)
anti-CD8 53-6.7 BV510 (Biolegend, 100751)
anti-CD8 53-6.7 BV605 (Biolegend, 100743)

anti-CD8 53-6.7 FITC (Biolegend, 100706)
anti-CD8 53-6.7 PE (Biolegend, 100708)
anti-TCRb H57-597 APC-Cy7 (Biolegend, 109220)
anti-TCRb H57-597 PerCPCy5.5 (Biolegend, 109228)
anti-IL2 JES6-5H4 APC (Biolegend, 503810)
anti-IL2 JES6-5H4 PE (Biolegend, 503808)
anti-IFNg XMG1.2 BV421 (Biolegend, 505829)
anti-TNF MP6-XT22 PE-Cy7 (ThermoFischer, 25-7321-82)
anti-Granzyme B QA16A02 APC (Biolegend, 372204)

Western Blot:
rabbit anti-ATG16L1 (EPR15638) (Abcam, ab187671)
mouse anti-GAPDH (6C5) (Sigma-Aldrich, MAB374).
IRDye 680LT Goat anti-Mouse IgG (H+L) (Licor, 926-680-70)
IRDye 800CW Goat anti-Rabbit IgG (H+L) (Licor, 926-322-11).

Identification of viable cells was done by fixable dead cell staining (Life Technologies, L34993 or L34957).
MitoSOX™ Mitochondrial Superoxide Indicator (Invitrogen) was used to detect mitochondrial superoxide
SnapTag labelling was done using cell permeable Snap-Cell substrates (New England Biolabs, NEB) in the following concentrations: 3 µM (Snap-Cell 647-SiR S9102S), 3 µM (Snap-Cell TMR-Star S9105S), 5 µM (Snap-Cell Oregon Green S9104S). SnapTag blocking was performed using 5 µM of unlabelled SnapSubstrate (Snap-Cell Block S9106S).

Validation

Antibodies were validated by using known negative and positive cell populations as controls. Some antibodies were previously validated by the manufacturer and information was extracted from their website.

Animals and other research organisms

Policy information about studies involving animals; ARRIVE guidelines recommended for reporting animal research, and Sex and Gender in Research

Laboratory animals

CD45.2 Omp25-SnapTagfl/fl mice were bred with CD45.1 Atg16l1fl/fl Ert2Cre OT-I mice expressing a TCR specific for OVA257–264 SIINFEKL peptide, and maintained as CD45.1 or CD45.1/2 mice in a C57BL/6 background. Host mice in adoptive transfer experiments were either B6.SJL.CD45.1 or C57BL/6 naïve mice. Six-to-sixteen-week-old mice were considered young and>100 week-old mice were considered aged.

Wild animals

The study did not involve wild animals.

Reporting on sex

For this study we used both male and female mice and did not observe any sex bias in our results.

Field-collected samples

The study did not involve samples collected from the field.

Ethics oversight

All animal work was reviewed and approved by Oxford Ethical committee and the UK Home office under the project licenses PPL30/3388 and P01275425.

Note that full information on the approval of the study protocol must also be provided in the manuscript.

Plants

Seed stocks

Report on the source of all seed stocks or other plant material used. If applicable, state the seed stock centre and catalogue number. If plant specimens were collected from the field, describe the collection location, date and sampling procedures.

Novel plant genotypes

Describe the methods by which all novel plant genotypes were produced. This includes those generated by transgenic approaches, gene editing, chemical/radiation-based mutagenesis and hybridization. For transgenic lines, describe the transformation method, the number of independent lines analyzed and the generation upon which experiments were performed. For gene-edited lines, describe the editor used, the endogenous sequence targeted for editing, the targeting guide RNA sequence (if applicable) and how the editor was applied.

Authentication

Describe any authentication procedures for each seed stock used or novel genotype generated. Describe any experiments used to assess the effect of a mutation and, where applicable, how potential secondary effects (e.g. second site T-DNA insertions, mosiacism, off-target gene editing) were examined.

Flow Cytometry

Plots

Confirm that:

☒ The axis labels state the marker and fluorochrome used (e.g. CD4-FITC).

☒ The axis scales are clearly visible. Include numbers along axes only for bottom left plot of group (a 'group' is an analysis of identical markers).

☐ All plots are contour plots with outliers or pseudocolor plots.

☒ A numerical value for number of cells or percentage (with statistics) is provided.

Methodology

Sample preparation	Blood samples used for kinetics analysis were obtained from the tail vein. Spleens and inguinal lymph nodes were harvested without perfusion. Single cell splenocytes were prepared by meshing whole spleens through 70 µm strainers using a syringe plunger. Lymph node single cell suspensions were prepared by meshing organs through 40 µm strainers using a syringe plunger. When cytokine production was assessed, CD8+ T cells within splenocytes were stimulated with 1 µg/ml of SIINFEKL peptide in the presence of Brefeldin A (Sigma-Aldrich) for 4-6 hours at 37°C. Identification of viable cells was done by fixable near-IR dead cell staining (Life Technologies). Erythrocytes were lysed by red blood cell lysis buffer treatment 5 min at room temperature. Surface stainings were performed for 20-30 min at 4°C. Specific CD8+ T cells were evaluated by incubation with SIINFEKL257-264-APC-Labeled or SIINFEKL257-264-BV421-Labeled tetramers (NIH Tetramer Core Facility at Emory University). All samples were washed and stored in PBS containing 2% FBS and 5mM of EDTA before acquisition.
Instrument	LSR II and Fortessa X20 (BD)
Software	BD FACSDiva (acquisition); BD Flowjo 10.10.0 (analysis)
Cell population abundance	For adoptive cell transfer experiments and in vitro assays, CD8+ T cells were isolated from spleens and inguinal lymph nodes using the EasySep Mouse CD8+ T cell Isolation Kit (naive or total population, StemCell), following manufacturer's instructions (purity >90%). We obtained 3-10x10^6 cells per mouse (3-10% of total splenic cells). For ex vivo analysis of splenic T cells from adoptive transfer experiments, 10% to 20% of the organ was used per staining. Frequencies of transferred cells within CD8 T cell populations varied across experiments.
Gating strategy	For all samples the following gating strategy was used: lymphocytes (SSC-A/FSC-A), exclusion of doublets (FSC-A/FSC-H), live cells (FSC-A/Live/Dead marker). To identify congenitally marked TCR transgenic CD8+ T cells, cells were gated for CD8 and for the congenic marker CD45.1 or CD45.2. First-daughter cells were identified by dilution of Cell Trace Violet.

☒ Tick this box to confirm that a figure exemplifying the gating strategy is provided in the Supplementary Information.
